# Measuring Well-Being in Sport Performers: Where are We Now and How do we Progress?

**DOI:** 10.1007/s40279-020-01274-z

**Published:** 2020-02-26

**Authors:** Samuel Giles, David Fletcher, Rachel Arnold, Arabella Ashfield, Joanna Harrison

**Affiliations:** 1 School of Sport, Exercise and Health Sciences, Loughborough University, Epinal Way, Loughborough, Leicestershire LE11 3TU UK; 2 Department for Health, University of Bath, Bath, UK; 3 Performance Lifestyle, English Institute of Sport, Manchester, UK

## Abstract

The importance of optimal well-being and mental health in elite athletes has received increasing attention and debate in both the academic and public discourse. Despite the number of challenges and risk factors for mental health and well-being recognised within the performance lifestyle of elite athletes, the evidence base for intervention is limited by a number of methodological and conceptual issues. Notably, there exists an increasing emphasis on the development of appropriate sport-specific measures of athlete well-being, which are required to underpin strategies targeted at the protection and enhancement of psychosocial functioning. Therefore, the purpose of this article is to review psychometric issues in well-being research and discuss the implications for the measurement of well-being in sport psychology research. Drawing on the broader literature in related disciplines of psychology, the narrative discusses four key areas in the scale development process: conceptual and theoretical issues, item development issues, measurement and scoring issues, and analytical and statistical issues. To conclude, a summary of the key implications for sport psychology researchers seeking to develop a measure of well-being is presented.

## Key Points

**Table:** 

The development of a sport-specific measure of well-being in sport performers is necessary to advance our understanding and more effectively support athletes’ health and performance.
To develop a measure of well-being in sport performers, scholars should address four key areas in scale development: conceptual and theoretical issues, item development issues, measurement and scoring issues, and analytical and statistical issues.
We have consolidated knowledge and understanding of the assessment of well-being across key psychometric issues and provided a robust platform for those who wish to assess athletes’ well-being.

## Introduction

Although athletes are, by definition, sport performers, they are fundamentally human beings whose physical, mental, and social health is reflected through their well-being and ill-being. As such, athletes’ holistic health is an integral aspect of who they are both as performers and as people. Human beings engagement in sport can contribute to or detract from the development of their well-being. In terms of physical well-being, it is well established that sports training enhances athletes’ neuromuscular, cardiovascular, and respiratory functioning, together with other physiological benefits such as improved immunology, metabolism, and sleep [1]. Turning to the mental benefits of sport participation, athletes typically develop a range of psychological skills that contribute to enhanced self-esteem, motivation, and resilience, that benefit not only their performance but also other aspects of their lives [2]. From a social perspective, organised sport activities provide a medium through which athletes communicate, develop relationships and collaboration, and foster a sense of belonging [3]. However, in contrast to these benefits, sport participation can undermine athletes’ well-being. Physical well-being is compromised when athletes become ill, injured, or overtrained due to their participation, or when they engage in unhealthy nutritional practices or substance abuse [4]. Mental well-being can be adversely affected by underperformance, pressure and expectations, burnout, and the development of maladaptive psychological symptoms and disorders [5]. Social well-being is threatened by an unsupportive environment, controlling practices, discrimination, harassment, bullying, hazing, abuse, conflict, and isolation [6–8].

One of the main features of an individual’s well-being is his or her mental health, which has been defined as a “state of well-being in which the individual realises his or her own abilities, can cope with the normal stresses of life, can work productively and fruitfully, and is able to make a contribution to his or her community” (p. 12) [9]. Hence, it is worth emphasising that mental health is not merely the absence of mental illness; rather, it spans a continuum from languishing to flourishing in life [10, 11]. This fundamental aspect of mental health and well-being has been recognised in the sport domain, which is increasingly promoting mental health as opposed to solely ameliorating mental illness in athletes [12–14]. Although the more positive aspects of mental health have attracted attention in recent years, Lundqvist [15] argued that in the sport psychology research literature “well-being is treated as an unspecific variable, inconsistently defined and assessed using a variety of theoretically questionable indicators” (p. 118). Indeed, it is conspicuous that no valid or reliable psychometric measure of athletes’ well-being exists [5, 15–18]. To circumvent this issue, researchers have typically employed proxy indicators of well-being, such as life satisfaction, affect, subjective vitality, psychological needs, self-esteem, and psychological distress. Although these concepts are necessary to understand well-being, they are individually not sufficient to provide a complete and accurate representation of the construct. Therefore, the continued and sole use of proxy indicators engenders conceptual ambiguity and, as a consequence, compromises understanding of the components of athlete well-being [15].

In contrast to the sport psychology literature, psychology more broadly has developed numerous psychometric measures of well-being. In a review of the literature, Linton et al. [19] identified 99 assessment instruments which collectively encapsulate six domains (viz., mental well-being, social well-being, physical well-being, spiritual well-being, activities and functioning, and personal circumstances). Some of the more prominent measures of well-being include the Personal Expressiveness Scale [20], WHO Quality of Life Brief Scale (WHOQOL-BREF) [21], WHO-100 Scale (WHOQOL-100) [22], Subjective Happiness Scale (SHS) [23], Mental Health Continuum Short-Form (MHC-SF) [11], Warwick–Edinburgh Mental-Wellbeing Scale (WEMWBS) [24], Flourishing Scale (FS) [25], BBC Subjective Well-Being Scale (BBC-SWB) [26], PERMA-Profiler (PERMA-P) [27], Scales of General Well-Being (SGWB) [28], and 14 item SGWB [29]. Available space precludes a detailed discussion of this literature, although it is worth highlighting that most scholars concur that well-being is a multifaceted phenomenon [30–32] and that attempts to measure well-being should reflect this complexity throughout the design, development, and delivery of psychometric questionnaires [33].

In this paper, we discuss the measurement of well-being in sport performers. More specifically, we review what we currently know about this topic in sport psychology and contemplate how psychometric advancements in psychology more broadly can inform progress in sport. Consolidating knowledge and understanding in this area will provide a robust platform for researchers and practitioners who wish to assess athletes’ well-being. From a research perspective, developing valid and reliable psychometric measures is necessary for advancing the evidence base in sport psychology [34–36]. From a practical perspective, accurately assessing athletes’ state-of-mind is important for effectively supporting their health and performance [37]. In line with Greenhalgh et al's. [38] recommendations, we adopted a narrative review approach because of the emphasis on scholarly summary along with interpretation and critique. Although other review methods may be more systematic in their approach, they are not without their limitations [39] and were deemed less appropriate for this literature review.

## Measuring Well-Being in Sport Performers

Attempting to measure psychosocial phenomena is a complex and difficult endeavour. Numerous intra- and inter-individual factors interact and fluctuate over time which means that assessing isolated constructs in a valid and reliable way is challenging. Human well-being is one such example. To better understand how to measure well-being in sport performers, we draw on DeVellis’s [40] work on scale development and organise our discussion around four psychometric areas: Conceptual and theoretical issues, item design and development issues, measurement and scoring issues, and analytical and statistical issues [41].

### Conceptual and Theoretical Issues

To measure well-being in sport, it is important for researchers to be clear about the exact nature and scope of the construct being assessed. The conceptualization of well-being has been extensively debated by scholars in psychology which has resulted in a variety of definitions derived from a range of conceptual and theoretical perspectives [42–45]. Despite the lack of a universally agreed definition of well-being, it is principally understood to encompass a combination of both hedonic and eudaimonic components [30, 33, 46, 47], which are crucial to thriving across multiple life domains [48]. The hedonic perspective is typically defined in terms of happiness which is achieved through the striving for rewarding and pleasurable experiences that reinforce positive feelings and satisfaction [49–51]. The eudaimonic perspective, as proposed by Aristotle (350 BCE/1985), focuses more broadly on the personal qualities and ways of life that promote living well. A central tenet to this perspective is the enactment of personal qualities that enable a person to live up to one’s personal potential in a manner that is consistent with their daimon (or ‘true self’). Grounded in eudaimonic principles, scholars have defined a variety of components (e.g. autonomy, personal growth, and purpose in life) that are used to study psychological well-being [52] and states of flourishing [53, 54]. Debate remains, however, regarding the extent to which particular components correspond to eudaimonia as articulated in the original philosophical works [55], as well as the extent to which these are empirically distinguishable from hedonic conceptions of well-being [56]. While a comprehensive review of the conceptual and theoretical discussion on this topic is beyond the scope of this paper, it is worth noting that this issue has provided a continual source of debate in the works of contemporary philosophers [57, 58], and eminent humanistic, clinical, and developmental psychologists [20, 53, 54, 59–64].

The study of hedonic well-being is often broadly equated with Diener’s [50] model of subjective well-being (SWB). With regards to measurement, there is general agreement that SWB comprises an affective component (i.e. the presence of positive emotions and the absence of negative emotions) and a cognitive component (i.e. evaluations of life satisfaction) [31, 50, 65]. Some of the most widely used measures to assess SWB components include the Satisfaction with Life Scale (SWLS) [66], Self-Anchoring Striving Scale [67], Positive and Negative Affect Schedule (PANAS) [68], and Scale of Positive and Negative Experience (SPANE) [25].

Turning to eudaimonic well-being, numerous conceptual models of measurement have been proposed that combine various components of psychological and social functioning and extend the notion of well-being beyond ‘feeling good’ as emphasised in the hedonic perspective [44, 69]. For example, Keyes and colleagues [10, 11, 70, 71] developed the mental health continuum model and a corresponding psychometric instrument (MHC-SF) that measures psychological and social functioning (termed psychological and social well-being), as well as positive affect and life satisfaction (termed emotional well-being), to provide an overall assessment of well-being ranging from languishing to flourishing. To illustrate the conceptual variance that exists, Table 1 depicts how various domains and components of well-being have been combined in prominent approaches to measurement. While there remains a lack of consensus regarding the conceptual structure of eudaimonic well-being, most scholars accept that measures of eudaimonic well-being are important because they provide an insight into the subjective experiences of individuals beyond that captured through assessments of life satisfaction and affect [72–75].

**Table 1:** Domains and components of example integrated measures of well-being

Domains and components	Integrated measures of well-being							
	MHC-SF	WEMWBS	National accounts of WB	FS	ESS	BBC-SWB	SGWB	PERMA-P
Emotional WB/SWB								
Emotional stability					●			
Positive emotions	●	●	●		●	●	●	●
Negative emotions			●			●		
Happiness						●	●	
Satisfaction with life	●		●					
Mental WB/PWB								
Autonomy	●	●	●			●		
Achievement			●		●	●		●
Competence		●	●	●	●		●	
Engagement			●	●	●		●	●
Environmental mastery	●							
Personal growth	●	●				●	●	
Purpose in life	●		●	●		●	●	●
Meaning	●		●	●	●		●	
Self-acceptance	●	●	●	●	●	●	●	
Congruence							●	
Vitality		●	●		●		●	
Optimism		●	●	●	●	●	●	
Resilience			●		●			
Social WB								
Positive relationships	●	●	●	●	●	●	●	●
Social acceptance	●							
Social coherence	●							
Social contribution	●			●				
Social growth	●							
Social integration	●		●			●		
Physical WB								
Physical health						●		●
Sleep						●		
Financial circumstances						●		
Access to health services						●		
Leisure						●		
Living circumstances						●		
Work circumstances			●			●		

*MHC-SF* Mental Health Continuum Short-Form [11], *WEMWBS* Warwick–Edinburgh Mental-Wellbeing Scale [24], *National Accounts of WB* National Accounts of Well-Being initiative [32], *FS* Flourishing Scale [25], *ESS* European Social Survey well-being module [46], *BBC-SWB* BBC Subjective Well-Being Scale [26], *PERMA-P* PERMA-Profiler [27], *SGWB* Scales of General Well-Being [28]

In extending this line of thought it is generally acknowledged that well-being is not simply the absence of psychopathology, but rather encompasses multiple domains, which in turn captures the complexity of optimal psychosocial functioning [76]. What has also become apparent is that well-established approaches to measurement are often founded on sound theoretical concepts and clearly specified characteristics that are common to the phenomenon of interest [77]. In sport psychology, scholars have increasingly sought to operationalise and theoretically scaffold the domains of well-being that characterise the experiences of sport performers [15, 78], whilst acknowledging that further explorative research is required to develop our understanding of its constituent parts [17, 78]. Furthermore, it is recognised that the establishment of a theoretical framework would serve several critical functions across psychometric development such as limit deficient operationalisations of the construct, improve content validity, and facilitate the specification and testing of nomological and construct validity [77]. In sport psychology, there are numerous examples of theory-driven measurement instruments adapted specifically to the sport context. For instance, Arnold and Fletcher [79] developed a taxonomic classification of organisational stressors in sport performers that, alongside other theories and evidence-based frameworks [80], informed the development of the Organizational Stressor Indicator for Sport Performers (OSI-SP) [81]. Similarly, the Self-Determination Theory [82] set the conceptual scope for several sport-specific measures such as the Behavioral Regulation in Sport Questionnaire (BRSQ) [83], Basic Needs Satisfaction in Sport Scale (BNSSS) [84], and Psychological Need Thwarting Scale (PNTS) [85].

It is also important that sport scholars distinguish well-being from related constructs to maintain conceptual clarity, as well as uphold discriminant validity and scientific legitimacy [77]. A lack of conceptual clarity has been an issue across psychology subdisciplines that has fuelled a proliferation of conceptual models and measures of well-being which has hindered the understanding of antecedents, correlates, characteristics, and consequences of well-being [19]. To illustrate, a recent review of self-report measures of well-being identified 196 facets of well-being across 99 psychometric instruments analysed [19]. In addition, the authors highlighted that there existed substantial heterogeneity in how well-being was understood. In view of this, it is suggested that future measures in this area distinguish well-being from related constructs to ensure that researchers’ focus remains on the true essence of well-being. Numerous psychometric tools have been established to assess constructs related to well-being, such as general self-efficacy (e.g. General Self-Efficacy Scale) [86], self-esteem (e.g. Self Esteem Scale) [87], personality (e.g. NEO-FFI) [88], and physical wellness [89]. Once a measure of well-being has been established for sport performers, scholars will then be able to explore and disentangle the relative contribution of various concepts and circumstances that influence an athlete’s overall state of well-being.

### Item Development Issues

Once the conceptual and definitional assumptions underpinning the development of a measure have been established, attention should focus on the design and construction of items (i.e. questions) that will form the instrument. As subjective well-being measures are primarily obtained via self-report methods, the responses provided by individuals will be dependent on how questions are phrased and interpreted [40]. Therefore, during the stage of item construction, it is important for sport scholars to consider the issues of item wording, comprehension, and interpretation.

As discussed previously, both hedonic and eudaimonic perspectives are central to the study of well-being; hence, to fully capture an athletes’ subjective experience, a measure of well-being should comprise a hybrid of components [44]. In recent psychometric guidelines on the measurement of well-being [90], it has been recommended that scholars consider including three broad categories of assessment—cognitive evaluations, affective states, and psychological functioning—in a tripartite fashion. However, in a more recent review, Huta and Waterman [42] argued that there existed a great deal of conceptual discrepancy in the terminology and operationalization of hedonic and eudaimonic definitions, and that well-being has interchangeably been studied as orientations (orientations, values, motives, goals), behaviours (behavioural content, activity characteristics), experiences (subjective experiences, emotions, cognitive appraisals), and functioning (indices of positive psychological functioning, mental health, flourishing). Specifically, the authors noted that hedonia and eudaimonia have sometimes been treated as asymmetrical terms which may introduce a confounding effect during the later stages of measure development if not recognised. In view of these discrepancies, it is apparent that sport scholars should pay careful attention to how well-being is operationally defined, and the terminology used in accordance to promote precision and consistency in the development of quality items [42].

Once a conceptual basis for measurement has been achieved and the array of components to be assessed has been explicitly defined, scholars should turn their attention to the construction of items. The first step in this process will require an extensive and diverse number of unbiased and meaningful statements to be developed [27, 40]. To facilitate this process, it is recommended that scholars consider adopting a systematic and inclusive approach towards item generation because it is commonly accepted that high item redundancy can be tolerated at this stage of psychometric development [40]. Furthermore, it is essential that the questions created are clearly justified and guided by theory grounded in athletes’ experiences to help ensure precision and an accurate representation of the components of interest [33, 40]. During this stage, a key issue that sport scholars will also need to consider is the impact of item wording effects on participant response. Although the psychometric literature regarding this issue is limited and mixed [90, 91], measures of affect and eudaimonic well-being (e.g. PERMA-P; SGWB) have tended to comprise of multiple questions per subscale to reduce item ambiguity effects (i.e. the random variation between individuals’ interpretations) [90]. However, the inclusion of multi-item subscales needs to be weighed against the possible issues of participant burden and common method variance effects [90, 92–94].

During the construction of items, researchers should also pay careful consideration to the phrasing and clarity of language used. To reduce measurement error and enhance the validity of responses, it has been recommended in the well-being and psychometric literature that scale items are kept relatively short, unambiguous, and that idioms or phrases that might not be understood by the target population are removed [95]. These issues are also relevant to measurement in sport, whereby scholars have identified that questions which lack specificity are likely to compromise accuracy [36, 96]. In addition, the inclusion of inversed and negatively worded items to limit response set biases is conceptually questionable in positive psychology research [27, 97], and needs to be weighed against the increased probability of method-induced biases [93, 98, 99]. To minimise such method effects, scale developers have either only included the positive components of well-being (e.g. WEMWBS; SGWB) or also included negative components (e.g. negative affect) which have then been scored separately to positive aspects and used to disrupt and test for response biases (e.g. PERMA-P).

To assess the quality of items developed and provide evidence for content validity, researchers should have the initial item pool reviewed by a group of experts that are knowledgeable in the field [40]. The composition of an expert panel to assess a measure of well-being in sport is likely to include sport performers, sport science and psychology practitioners, performance lifestyle advisors, coaches, psychometric development specialists, and sport psychology researchers. Once a panel of experts has been formed, it is recommended that formalised rating procedures are developed to evaluate the quality of the generated content for relevance, representativeness, specificity, and clarity [100]. In turn, the expert feedback will (a) provide evidence for whether the content theorised to be related to the construct of athlete well-being and the actual content of the psychometric match, and (b) highlight any potential threats to content validity such as construct irrelevant content and construct under-representation [100]. In addition, sport scholars may also want to consider employing methods to assess participant response processes such as think-aloud protocols [100–103]. Specifically, a variety of practical techniques may be used by scholars either concurrently (i.e. as the participant responds to questions) or retrospectively (upon completion of questions) to examine the mechanisms through which participant response occurs [104–107]. Put simply, for a psychometric instrument of well-being in sport to be accurate, sport scholars need to be confident about the extent to which the psychological processes hypothesised to be under investigation match the processes that respondents actually engage in when responding to items [103, 108, 109]. Although methodological progress remains frugal in this area, and there exists much debate regarding the optimal approach and implementation of these techniques [100, 110], some psychometricians have advocated that this step be considered when feasible in the development of a psychometric instrument [100].

Items developed for a questionnaire need to be relevant and specific to those individuals who will be responding to them to produce accurate and meaningful data. At present, the current emphasis of psychometrics in well-being research resides in the development of context-free measures that enable comparisons across different populations and settings [111, 112]. However, the reliance on global measurement instruments is unlikely to capture a complete rendering of well-being experiences that are idiosyncratic to athletes [15]. Drawing on the related occupational and organisational literature, domain-specific measures of well-being have been deemed necessary to “capture the subtleties, complexities, and variation of employees’ cognitive and affective experiences” (p. 446) [113]. With regards to sport, research suggests that sport performers may differ in their ability to introspect, respond to self-report measures, and could find it challenging to answer broad questions about their well-being [89, 96]. A measure that includes both general and specific items attuned to the unique context of sport, competitive standard of athletes, and psychosociophysiological factors, is likely to yield the most accurate and sensitive assessment of well-being in athletes.

### Measurement and Scoring Issues

In conjunction with the construction of questions, sport scholars should remain mindful of the methodological issues that relate to the measurement and scoring of these items. Although the focus of this paper is on the measurement of subjective accounts, it is important that researchers are aware that a combination of both subjective and objective assessments will likely capture the most comprehensive picture of human well-being [30, 114, 115]. This aligns with the recommendations outlined in the OECD Better Life Initiative [116, 117], work conducted with employees in organisations [118, 119], and with approaches that have been implemented to index the well-being of citizens nationally [32, 120]. Scholars who have endorsed objective approaches to the measurement of well-being have often used objective list accounts based on assumptions about basic human needs and rights, and indicators such as education, career success, and material comforts [30, 121, 122]. Whereas subjective accounts of well-being assess the views of individuals, which is important, as they provide an insight into the “picture of well-being that is grounded in people’s preferences, rather than in a priori judgements about what should be the most important aspects of well-being” (p. 183) [90].

In the majority of research, subjective well-being is often captured through retrospective assessments which request respondents to recall their experiences over a pre-specified reference period (e.g. “these days”, “nowadays”, and “the last two weeks”) [123]. The use of self-report methods such as questionnaires or diaries is merited because they capture a snapshot of well-being that is grounded in human perception, whilst remaining a cost effective and practical method of data collection [124]. There is also evidence to suggest that they correlate reasonably with other indices of well-being such as biological measures, memory, and experience sampling measures [31, 125]. However, a major limitation of self-report measures is that response biases and survey design features can distort the recollection and aggregation of reported experiences [123].

In view of the limitations of standard self-report methods, some scholars have advocated for the use of experience measures designed to capture momentary data. For instance, the experience sampling method (ESM) is recognised as the gold standard in capturing affect in day-to-day life as it records data from participants in real-time at random intervals about current engagements and feelings [126, 127]. An alternative approach is the day reconstruction method (DRM) which requests participants to reconstruct their experiences of the preceding day and is designed to limit participant burden associated with ESM [128, 129]. The advantages of both ESM and DRM are that they are able to systematically portray a more fine-grained picture of SWB experiences (e.g. positive emotions), whilst minimising biases associated with the accuracy of recall. Furthermore, researchers have suggested that the versatility of these methods could also be extended to capture more momentary eudaimonic facets of well-being, such as engagement [130].

A further measurement and scoring issue that sport scholars should consider relates to what is actually being measured by questionnaires. Drawing from the psychometric literature on well-being [90], it is apparent that scholars should determine the structural dimensions of the components that measurement will be based upon, which could include arousal (high vs low), prevalence (frequent vs infrequent), intensity (high vs low), specificity (“global” context free vs domain specific vs facet specific), stability (trait vs state), temporality (past vs present), typicality (typical vs atypical), and valence (pleasant vs unpleasant). Furthermore, it is recommended that a variety of these dimensions are considered in the design of suitable response indicators. To illustrate, in the measurement of affect, scholars have assessed the subjective experience of positively or negatively valanced feelings including lower activation feelings (e.g. calm, pleasant, relaxed) and higher activation feelings (e.g. active, energetic, excited). Scholars have also identified state and trait differences in the assessment of affect, and more recently started to examine variances across frequency, intensity, and the typicality (e.g. experience of unpleasant happiness “feeling fatigued by training but happy to have achieved a desired training goal”) of emotional experiences [131]. By comparison, the evidence base underpinning the measurement of eudaimonic components across multiple dimensions (e.g. frequency, intensity) is less understood, and more research is needed which examines the properties and performance of relevant psychometric instruments.

Once the dimensions which are to be measured have been decided, researchers should consider and select the most appropriate response format and scale characteristics. This is an important step because decisions made at this stage will influence the validity, reliability, and comparability of responses [132]. Although a number of well-established formats exist (e.g. the semantic differential, visual analogue scales) [40], subjective measures of well-being have typically utilised Likert scales (e.g. SWLS) [66]. When responding in this format, participants are asked to provide a judgement (e.g. how often did you feel?) in response to a declarative statement that pertains to a specific indicator of well-being (e.g. that your life has a sense of purpose?). If sport psychologists opt for a Likert format, they will also need to carefully consider a variety of response scale characteristics [40, 133], including scale polarity (bipolar vs unipolar), scale length (i.e. optimal number of response categories or range between minimum and maximum value), the inclusion of a neutral point, scale labelling (e.g. selection of verbal and numerical labels, length and amount of information conveyed, use of quantifier labels, number of fixed reference points), scale ordering (e.g. negative-to-positive, 0-to-positive), scale symmetry (symmetric vs asymmetric), and visual presentation of the scale (e.g. types of visual response requirements, use of illustrative formats, and the scales layout display). Following this stage of development, scholars will also need to decide on the mode (e.g. self-administered questionnaires, pen-and-paper interviewing, telephone interviews and computer-assisted telephone interviews) [90] by which questions will be disseminated. Increasingly scholars have examined the impact of psychometric design on participant response tendencies and data quality. Interested readers are encouraged to consult key texts for updated guidelines in the psychometric literature [40, 133–135] and well-being literature [90].

After respondents have completed well-being questionnaires, sport psychologists will need to consider the issue of how best to score and present information on a wide array of subjective well-being facets. Additive scoring methods have often been used which sum the item scores to produce an overall composite indicator for a subscale and / or construct (see, e.g. WEMWBS; FS; PERMA). However, the real-world utility of a summary score of individual well-being has been questioned by scholars in broader psychology, as illustrated in the following quote:

We suggest that in presenting individual or group results, the multidimensional structure of the measure should be retained, rather than condensing responses to a single flourishing score … Further, while a single overall flourishing score might provide a global indication of well-being, it obscures potentially meaningful variation amongst the domains. For instance, if a person scores particularly low in relationships, interventions might target strategies for building social connections (p. 21) [27].

Instead, a ‘dashboard approach’ has been proposed as a useful method to convey information, whereby the scores of each component are averaged to produce several distinct scores that illustrate the multiple ways in which well-being is cultivated [30, 54]. Furthermore, as an alternative to additive methods, scholars have discussed the merit of prescriptive and categorical approaches towards scoring that are based on thresholds to determine the prevalence of well-being states within a population [90]. Such an approach is similar to cut-off points that have often been used in measures of ill-being (i.e. Generalised Anxiety Disorder Assessment, Patient Health Questionnaire) [136, 137] to identify and assess psychological distress. Although such approaches are useful in presenting the distribution of data within a single figure, caution is advised in the selection of thresholds which must be both data-driven and theoretically meaningful [33, 90]. From a scoring interpretation perspective, researchers should also consider whether the highest levels of well-being are always desirable, particularly given the potentially complex relationship with athlete performance at the highest level [138].

### Analytical and Statistical Issues

Once researchers have confirmed measurement and scoring approaches, they will need to consider several analytical and statistical issues. Initially, sport psychologists seeking to measure well-being should determine the characteristics of the sample criteria regarding the competitive standard, number of participants to be recruited, as well as the frequency with which participants are assessed. It is assumed that aspects of subjective well-being will differ between recreational athletes in the general population and elite sport performers [17, 139], particularly when the unique physical and psychosocial risk factors inherent in the lifestyle of high performing individuals are considered [5]. Therefore, it is important that the ability of the sample (e.g. Olympic, recreational) is clearly defined and selected to prevent the emergence of scale reliability issues [40]. In addition, scholars will need to consider the size of the sample that questions will be administered to during the validation stage of measure development. As there are no accepted standards for the sample size required to adequately test the properties of psychometric instruments, the recommendations for estimating sample size in psychometric validation studies vary markedly [140, 141]. For instance, some psychometricians have recommended the use of absolute minimum participant numbers (e.g. 100) [142] or arbitrary benchmark criteria (e.g. 50 = very poor, 100 = poor, 200 = fair, 300 = good, 500 = very good, and 1000 = excellent) [143], whereas others have advocated that to calculate sample size scholars should consider multiple issues such as the ratio of number of participants to number of variables [144], the level of communality between variables [141, 145], and the ratio of variables to factors [141, 145, 146]. More recently, Myers et al. [147] provided further direction on the issue for scholars in sport and exercise science and the importance of sample size determination and power estimation in psychometric development research. Finally, sport scholars should consider sampling a cohort of athletes across different points in time which would reduce error and correct for attenuation and the true strength of relationships estimated between items [148].

Before scholars can measure the well-being of sport performers they may need training in a number of statistical software packages (e.g. SPSS, MPlus, R, EQS) that can be used to examine the structural properties of a psychometric instrument. Specifically, these software programmes can be used by scholars to assess: (1) the prerequisite statistical assumptions of the data (e.g. distribution of data), and (2) the factor structure of the measurement model by examining the validity of participant responses (e.g. scores) and the relationships between latent variables whilst accounting for measurement error [149]. Typically, exploratory factor analysis (EFA) is used in the early stages of psychometric development or modification to examine the communality between items and how items can be collated to form the “best fit” that represents relationships between observed variables (e.g. perceived purpose in life, experienced positive affect, and perceived belonging) and potential underlying latent variables (e.g. psychological well-being and social well-being) [150]. Once scholars have established a clear hypothesis about the underlying factor structure, more advanced factor analytical methods have often been used to provide further evidence for structural validity, including confirmatory factor analysis (CFA) and, more recently, exploratory structural equation modelling (ESEM). Specifically, the idea behind CFA is to test a stringent hypothesis that certain items are tapping a latent factor without any cross-loadings onto other latent factors. This model is, therefore, considered to differ from the EFA model because scholars using CFA apply a set of constraints based on a priori hypothesis regarding the number of latent factors and the relationships between items that describe them [149]. By comparison, in ESEM the only a priori information required to run analysis is the number of latent factors; all other parameters are then freely estimated [151, 152]. A detailed description of EFA, CFA and ESEM is beyond the scope of this review; however, interested readers are encouraged to consult Field [150], Geiser [153], and Heck and Thomas [154] for more information, as well as previous research that has implemented some of these methods in sport [81, 85, 155] and outside of sport [27, 28].

Although the statistical methods discussed above have often been used in the development of psychometric assessment tools, it is important for scholars interested in measuring athlete well-being to be aware of a number of critical considerations. Firstly, EFA has traditionally been used prior to CFA; however, scholars should be aware that CFA does not necessarily provide verification or confirmation of EFA findings, or need to be preceded by EFA [156]. It is, therefore, important for scholars to consider the rationale as well as the appropriate methods for implementing the chosen factor analytic techniques [156]. Secondly, a possible overreliance on traditional factor analytical techniques (and related fit indices) to examine the factor structure of responses to questionnaires has been acknowledged in sport and exercise psychology [149, 156]. Sport psychologists seeking to measure well-being will need to weigh up the conflicting need for validity based on content with the validity based on the evidence of the factor structure. Where appropriate, scholars might want to consider more advanced techniques (e.g. ESEM) to establish the statistical properties of a psychometric tool [151, 157, 158] and incorporate method factors that might enable the proper estimation of ‘good fitting’ subscales with several indicators [149].

Whilst deciding on the optimal length of the scale, scholars need to account for the reliability coefficients (i.e. alpha values) which are influenced by both the scale length and extent of covariation among items. In a review of the measurement literature, Saw et al. [89] recommended that psychometric developers carefully consider the breadth of components included and the number of items in a measure as “these two issues are often the reason that sports programmes tend to incorporate elements of established measures into their own brief custom self-report measure rather than adopting an existing measure from the literature” (p. 10). Drawing on research in broader psychology, it is interesting to note that two validated and brief well-being scales, the Short Warwick–Edinburgh Mental-Wellbeing Scale (SWEMWBS) [159] and Flourishing Scale (FS) [160] incorporate only single-item constructs, despite researchers and psychometricians maintaining that it is desirable to use several good items as indicators of each feature [46, 90, 98]. With regard to the implications for sport, it is crucial for psychologists to reflect on the trade-off between shorter more practical measures on the one hand, and longer and typically more reliable scales on the other.

A further analytical and statistical consideration concerns the accuracy of the measure and ensuring validity throughout the design and development process. To elaborate, when implementing well-being measurements within a sport context, researchers must be confident about the extent to which questionnaires actually capture the underlying concept that they purport to measure [90, 150]. Despite a consensus supporting the extent to which measures of both life evaluation and affect capture valid information in general [125, 160], the evidence base for eudaimonic measures remains less clear and requires further attention [90]. With this in mind, it is critical for scholars to account for a number of confounding variables that are unrelated to the actual experience of subjective well-being and limit respondent error [90, 150]. Bradburn, Sudman, and Wansink [161] outlined four cognitive factors (i.e. recall error and memory failure, question miscomprehension, lack of knowledge, and lack of motivation) that are associated with the increased risk of response biases and heuristics (e.g. sub-conscious cognitive short-cuts). As a result, individual responses to well-being surveys are sensitive to specific survey design factors such as question order effects, the content and wording of preceding questions, and the mode of survey delivery [162]. The precision of responses is also likely to be limited through more transient and occasional factors, including one-off or circumstantial events (e.g. competition selection outcomes), days of the week, or changes in momentary mood prior to the survey [163]. Furthermore, cues from the wider social context should also be considered by scholars when measuring well-being. For instance, in a sport context, athletes may draw on information pertaining to social norms, past experiences, and future hopes which could induce socially desirable responses and impression management biases (e.g. ‘faking good well-being’ to imply coping when close to a selection) [96]. Therefore, it is important for sport scholars to consider the influence of possible confounding variables in both the design of a questionnaire which minimises the influence of self-report measurement design, and the individual and situational factors which may influence the ability to obtain meaningful, accurate, and consistent data from athletes [96].

## Concluding Remarks

Although sport psychology has been slow to consider the measurement of well-being in sport performers, it is clear that psychometric advances in psychology more broadly can inform progress in this area. To this end, we have consolidated knowledge and understanding of the assessment of well-being across a range of scale development issues and the implications for sport psychology. The main messages to emerge from this discussion are presented in Table 2 and provide a robust platform for researchers and practitioners who wish to assess athletes’ well-being. As noted at the outset of this paper, athletes are not just performers, they fundamentally are people. As such, although performance enhancement is an inherent feature of sport, so to should be the optimisation of athletes’ health and well-being. Indeed, athletes train and compete within complex organisational structures that have a collective ethical and legal obligation to safeguard athletes’ welfare. This duty of care extends beyond athletes’ physical health to include their psychological and social well-being. This is perhaps no more apparent—and at risk—than in elite level sport where an insatiable need for success can be at odds with living a balanced lifestyle. We argue that policy decisions at the government, corporate, and organisational levels throughout sport should be more heavily influenced by issues related to athlete well-being. Current policy is dominated by participation and performance agendas and pays limited attention to athletes’ well-being, and how it can be assessed and enhanced. Our clarion call for sport policymakers echoes Diener and Seligman’s [164] remarks that “well-being should become a primary focus of policymakers, and that its rigorous measurement is a primary policy imperative” (p. 1).

**Table 2:** Main psychometric issues and recommendations for measuring well-being in sport performers

Psychometric issues	Main recommendations
Conceptual and theoretical	In the development of a measure of well-being in sport performers it is crucial that sport psychology researchers ensure that the structure of the instrument is theoretically grounded, contextually relevant, and empirically robust It is important for sport scholars to reach a consensus on the definition of well-being in sport performers. In view of the literature reviewed, well-being is best conceptualised as a dynamic and multi-dimensional state that further comprises of a variety of sub-component indicators The distinction between influencing factors, states of well-being (i.e. characteristics), and consequences of well-being (i.e. outcomes), is often blurred in the psychometric literature. Therefore, scholars should distinguish well-being from several related phenomena and provide a clear justification of the specific constructs or relationships among constructs that they intend to measure The establishment of a theory-driven definition and model of athlete well-being can provide scholars with a greater understanding of well-being, the implications of well-being related concepts and their findings
Item design and development	For subjective measures to be effective in obtaining information on the well-being of athletes, sport scholars need to consider several item development issues such as wording, comprehension, and interpretation of questions In accordance with a multi-dimensional view of well-being, it is recommended that scholars incorporate evaluations of cognition, affect, and psychological functioning—in a tripartite fashion To limit measurement error and enhance validity, careful considerations should be paid to the phraseology of items, to ensure that these are kept short, unambiguous, and easily understood. Scholars should also consider the specificity of items included, as well as the implications of negatively and positively weighted items To assess the quality of questions developed and to ensure the content validity of the scales constructed, it is recommended that sport scholars have the initial item pool reviewed by a diverse expert panel
Measurement and scoring	It is recognised that a combination of both objective and subjective assessments are required to provide an overall picture of human well-being. Yet, subjective measures are considered fundamental, in that they provide an overview of well-being that is grounded in people’s preferences, rather than in a priori judgements about what should be the most important aspects of well-being It is important that sport scholars consider the variety of subjective measurement approaches designed to assess well-being (e.g. self-report, experience sampling methods etc.) and select the method that is conceptually aligned with their research objectives The use of self-report methods are justified as they capture a snapshot into the well-being experiences of athletes whilst also remaining a practical and cost effective method of data collection To capture a complete rendering of subjective well-being, it is recommended that sport scholars combine a variety of dimensions into their measure (e.g. dimensionality, polarity, valence) to reflect the breadth and depth of well-being components It is recommended that sport scholars carefully consider the response format to be selected, the length of response scales, as well as the presentation of response categories, so that the need to capture as much meaningful variation is balanced against minimising respondent burden and frustration With respects to scoring, a ‘dashboard approach’ is suggested as a useful method to convey information, whereby the scores for each component indicator are averaged to produce several distinct domain-specific scores that illustrate the multiple ways in which well-being is achieved During the validation stage of psychometric development, it is important that sport scholars carefully consider the number of participants, frequency of participant assessment, as well as the ability level of participants recruited
Analytical and statistical	Sport scholars should acknowledge several analytical and statistical issues when developing a psychometric assessment tool. Specifically, scholars are encouraged to critically consider the approaches that have been used traditionally to examine the psychometric properties of measurement models (e.g. EFA and CFA) against more advanced methods (e.g. ESEM) When deciding on the optimal length of the scale to be used in sport, it is crucial that sport psychologists consider and reflect on the trade-off between shorter more practical measures on the one hand, against longer and more reliable scales on the other Sport scholars must be confident on the extent to which the scale captures the underlying concepts that they purport to measure. Therefore, it is critical for researchers to account for various confounding variables that are not related to the actual experience of subjective well-being and limit respondent error. These include issues relating to the design of the questionnaire, as well as situational factors (e.g. timing in competitive season, environment and location, people distributing the questionnaire and relationship with the sport performers) which may influence the acquisition of meaningful, accurate, and consistent data from athletes

As alluded to above, those involved in all aspects and levels of sport make decisions that should be guided by sound ethical principles, such as responsibility, respect, and dignity. For example, some ethical issues that may present to individuals operating in sport with regards to the measurement of well-being could involve the collection, storage and control, access, and use of confidential and sensitive personal data (i.e. well-being scores). Accordingly, a shared collective duty for the welfare of athletes, both within the sports in which they train and compete and beyond them, should be acknowledged.^1^ In addition to this ethical obligation, there are also legal imperatives^2^ relating to athletes’ health and well-being. This legal obligation is essentially encapsulated by the tort of negligence, which refers to a legal wrong that is suffered by someone at the hands of another who fails to take proper care to avoid what a reasonable person would regard as a foreseeable risk. The confluence of the ethical and legal obligations for human welfare, together with reciprocal link between well-being and performance [165, 166], has led to the emergence of a number of regulatory bodies and policy developments^3^ [164, 167]. Although the sport sector has been slow to recognise such developments, initiatives such as the UK Government’s 2015 Mental Health Charter for Sport and Recreation [168], the UK Government’s 2017 Duty of Care in Sport Review [169], the UK Government’s 2018 Government Mental Health and Elite Sport Action Plan [170], UK Sport’s 2018 Mental Health Strategy for the High Performance System [171], Sport New Zealand’s 2018 Elite Athletes’ Rights and Welfare review report [172], and the International Olympic Committee’s 2019 consensus statement on Mental Health in Elite Athletes [173] represent promising policy and strategic level advances in the sport context.

Despite these regulatory, policy, and strategic advances, there is little evidence-based understanding of athlete well-being in sport. We propose that the development of a sport-specific measure of well-being in sport performers is necessary to progress what is known in this area and more effectively support athletes’ health and performance. Moreover, such a measure, together with other indicators of well-being (e.g. athlete and staff surveys, interviews and appraisals, psychophysiological and neurological assessment, clinical and psychiatric assessment, physical health and medical screening, team audits, human resource data, performance and attainment metrics), should inform the development of athlete well-being and mental health screening processes within sports, benchmarking against other sports and broadly comparable sectors (such as the armed forces and construction industry), and national and international sport governing bodies' well-being indexes and databases. Such developments are crucial to provide a more rigorous and robust approach to assessing, monitoring, and evaluating not only athletes’ health and well-being, but also all those who operate in sport environments (e.g. coaches, managers, directors, officials, administrative and support staff, fans, etc). Systematic and periodic assessment of well-being will offer those operating in sport, and policymakers who influence sport, a much stronger foundation on which to base their decisions.

## Footnotes

1 The ethical obligation and duty of care for the welfare of athletes can be traced back to the philosophy of the Olympic Games, Olympism. Among the principles of Olympism is that every individual must have the possibility of practising sport, without discrimination of any kind and in the Olympic spirit, which requires mutual understanding with a spirit of friendship, solidarity and fair play [174]. During the early twentieth century, several sport associations (e.g. Amateur Athletic Union, Professional Golfers’ Association, Professional Footballers’ Association) were formed to support athletes’ development and participation in several sports [175]. As sport has modernised, its governance and organisation have become more complex, and athletes’ welfare and well-being are considered in diverse ways. For example, in the 1950s and 1960s the major North American sports formed players’ associations [175]; in 1985, the Canadian Olympic Athlete Career Centre was established in Canada [176]; in 1989, the Lifeskills for Elite Athletes Program was launched in Australia [176]; in 1999, the Athlete Career and Education Program UK was set up in the United Kingdom [177]; and in 2017, the UNI Global Union published the World Player Development, Wellbeing, Transition and Retirement Standard [178].

2 In the UK, for example, the Health and Safety at Work Act (1974) imposes a legal duty on employers to ensure, so far as is reasonably practicable, the health and safety of their employees and others who are not employees in places of work. Although initially this legislation focused on physical health and safety there has been a shift in the interpretation to include psychological and social welfare [179–183]. Other legislation, such as the Management of Health and Safety at Work Regulations (1999), the Protection from Harassment Act (1997), and the Equality Act (2010), reinforces that those in positions of responsibility in organised sport have a duty to safeguard not only athletes’ physical health and safety but also their broader welfare and well-being.

3 For example, the HSE regulates workplace health and safety in the UK and expects organisations to carry out appropriate risk assessments. From a psychosocial perspective, the HSE developed the management standards programme, which is essentially a three-phase process based on a risk assessment to identify potential stressors in the workplace and associated targeted interventions [184, 185]. More recently, the UK National Institute for Health and Care Excellence (NICE) [186] has published evidence-based guidelines for managers on how to promote employee well-being, and the UK Government commissioned independent review of thriving in work [187] has proposed standards for better supporting the mental health of all people in employment.
